# The Role of Phosphate Group in Doped Cobalt Molybdate: Improved Electrocatalytic Hydrogen Evolution Performance

**DOI:** 10.1002/advs.201903674

**Published:** 2020-04-22

**Authors:** Siyu Zhao, Jasper Berry‐Gair, Wenyao Li, Guoqiang Guan, Manni Yang, Jianwei Li, Feili Lai, Furio Corà, Katherine Holt, Dan J. L. Brett, Guanjie He, Ivan P. Parkin

**Affiliations:** ^1^ Christopher Ingold Laboratory Department of Chemistry University College London 20 Gordon Street London WC1H 0AJ UK; ^2^ School of Materials Engineering Shanghai University of Engineering Science Shanghai 201620 China; ^3^ Electrochemical Innovation Lab Department Chemical Engineering University College London London WC1E 7JE UK; ^4^ State Key Laboratory for Modification of Chemical Fibers and Polymer Materials College of Materials Science and Engineering Donghua University Shanghai 201620 China; ^5^ School of Chemistry University of Lincoln Joseph Banks Laboratories Green Lane Lincoln LN6 7DL UK

**Keywords:** density functional theory, electrocatalysts, hydrogen evolution, in situ ATR‐IR

## Abstract

The hydrogen evolution reaction (HER) is a critical process in the electrolysis of water. Recently, much effort has been dedicated to developing low‐cost, highly efficient, and stable electrocatalysts. Transition metal phosphides are investigated intensively due to their high electronic conductivity and optimized absorption energy of intermediates in acid electrolytes. However, the low stability of metal phosphide materials in air and during electrocatalytic processes causes a decay of performance and hinders the discovery of specific active sites. The HER in alkaline media is more intricate, which requires further delicate design due to the Volmer steps. In this work, phosphorus‐modified monoclinic *β*‐CoMoO_4_ is developed as a low‐cost, efficient, and stable HER electrocatalyst for the electrolysis of water in alkaline media. The optimized catalyst shows a small overpotential of 94 mV to reach a current density of 10 mA cm^−2^ for the HER with high stability in KOH electrolyte, and an overpotential of 197 mV to reach a current density of 100 mA cm^−2^. Combined computational and in situ spectroscopic techniques show P is present as a surface phosphate ion; that electron holes localize on the surface ions and both (P—O^1−^) and Co^3+^—OH^−^ are prospective surface active sites for the HER.

## Graphical abstract

A P‐doped Ni foam/CoMoO_4_ electrocatalyst is successfully prepared by a facile hydrothermal‐annealing method. Optimized P‐doped Ni foam/CoMoO_4_ shows excellent hydrogen evolution reaction (HER) activity (94 mV@10 mA cm^−2^) and remarkable stability in 1 M KOH. A combined in situ spectroscopic and computational study shows the mechanism of P doping and active sites for the catalyst.

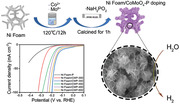

## 1 Introduction

With rising global energy demands, much attention has been paid to the development of clean and renewable energies.^[^ ^1^ ^]^ Hydrogen is one of the most promising fuels as a replacement of fossil fuels.^[^ ^2^ ^]^ Electrolysis of water is a chemically straight‐forward way to produce hydrogen at large‐scale and would allow the use of surplus energy generated via renewable sources to evolve H_2_ in high purity. Platinum possesses an almost thermal‐neutral hydrogen adsorption free energy $\Delta G_{H}$. Thus, Pt requires a small overpotential for hydrogen evolution reaction (HER) and is known as the most efficient HER electrocatalyst.^[^ ^3^ ^]^ However, the high cost limits the wide application of noble metal catalysts. Therefore, the design and synthesis of high‐performance and low‐cost electrocatalysts is a stringent requirement to enable market uptake.

Transition metal elements, for example Ni,^[^ ^4^ ^]^ Co,^[^ ^5^ ^]^ Mn,^[^ ^6^ ^]^ Fe,^[^ ^7^ ^]^ and V^[^ ^8^ ^]^ in different monometallic and bimetallic compounds, such as oxides,^[^ ^9^ ^]^ carbides,^[^ ^10^ ^]^ nitrides,^[^ ^11^ ^]^ sulfides,^[^ ^12^ ^]^ and phosphides^[^ ^13^ ^]^ have been explored as alternative HER electrocatalysts. This is due to their abundance, low cost, and potentially tuneable electrocatalytic properties. Metal phosphides were demonstrated as one of the most effective HER catalysts due to their high electronic conductivity and optimized H* intermediate adsorption energy from P^3−^ in acidic media. Similar to the steps in acidic electrolyte, HER pathways in alkaline media are comprised of Volmer–Heyrovsky or Volmer–Tafel processes. However, the use of alkaline media for HER is more challenging, as a more complicated reaction during the Volmer step is required for the initial dissociation of water in alkaline media. In addition, the instability of metal phosphides during both storage and reaction conditions in both acidic and alkaline media requires further attention. Atomic level detail on the specific active sites is critical for rational optimization of catalytic performance.^[^ ^14^ ^]^

A range of modification methods which include transition metal/carbon hybrids,^[^ ^13^, ^15^ ^]^ defect/surface engineering,^[^ ^16^, ^17^ ^]^ electronic, and structural modification by combining different metal species^[^ ^18^, ^19^ ^]^ have been devoted to improve the HER performance. In a typical HER “volcano plot” for metals, Co and Mo possess near neutral hydrogen adsorption energies, which are close to that of Pt and are regarded as promising elements for efficient HER.^[^ ^1^ ^]^ Based on this understanding, Co and Mo based materials, such as Co_3_O_4_,^[^ ^20^ ^]^ CoP,^[^ ^21^ ^]^ and MoP,^[^ ^22^ ^]^ have been intensively studied during the electrocatalytic process of HER. Nevertheless, most single metal‐based electrocatalysts have a limit in their intrinsic activity as it is difficult to balance the adsorption and desorption of intermediates by tuning their chemical composition. Combining two or more transition metals in electrocatalysts has been demonstrated as an efficient way to obtain the desired $\Delta G_{H}$ for the HER.^[^ ^23^, ^24^ ^]^ For example, bimetal nitrides, that is, Ni*_x_*Co_3‐_ *_x_*N, were demonstrated to have an optimized energy for absorption/desorption of intermediates compared to Co_3_N or Ni_3_N counterparts during oxygen evolution and oxygen reduction processes.^[^ ^25^ ^]^ In addition, Zhao et al. found that the HER performance bimetallic Co_4_NiP was better than monometallic CoP and Ni_2_P electrocatalysts.^[^ ^26^ ^]^

Stable alternatives to binary metal phosphides as HER electrocatalysts in alkaline electrolyte were first reported by Chen et al.,^[^ ^27^ ^]^ who used P‐doped CoMoO_4_ structures that utilize multiple active sites in the Co, Mo, and P phase space. From computational studies they discovered that P decreases the hydrogen adsorption free energy and increases the water dissociation energy. However, a systematic research of P substitution in CoMoO_4_ structures and their function to give the increased performance is not yet available. In this work, the synthesis‐structure‐performance relationship is carefully investigated based on a series of P‐modified CoMoO_4_ (CMP) nanosheets supported on Ni foam. The results show that materials phosphatized at 350 °C have the lowest overpotential of 94 mV to reach a current density of 10 mA cm^−2^. Furthermore, a combined X‐ray photoelectron spectroscopy (XPS), in situ ATR‐IR and computational study revealed that MoO_4_^2−^ was substituted by PO_4_^3−^ during P‐doping process where the charge is compensated by a hole localized on the phosphate oxygen ion exposed on the surface, which would lead to an enhanced HER performance. And both (P—O^1−^) and Co^3+^—OH^−^ are prospective surface active sites for the HER.

## 2 Results and Discussion

**Figure 1:**
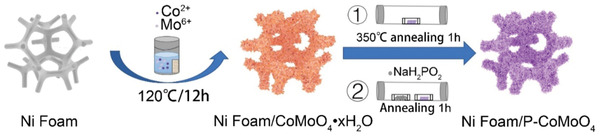
Schematic illustration for the preparation of Ni foam/P‐CoMoO_4_ catalyst.

The CoMoO_4_‐based materials in this work were synthesized via a two‐step strategy, schematically illustrated in **Figure** **1**. The first step involved a facile hydrothermal growth of CoMoO_4_·*x*H_2_O (CMO‐precursor) on the nickel foam (Ni foam/CMO‐precursor) by using Co(NO_3_)_2_·6H_2_O and Na_2_MoO_4_·2H_2_O as the Co and Mo sources, respectively. The as‐obtained compounds were transformed into CoMoO_4_ (CMO) by annealing in a tube furnace at 350 °C for 1 h. Then P was incorporated into the crystal structure of CMO by PH_3_, which was released by decomposition of 500 mg NaH_2_PO_2_·H_2_O above 200 °C. Different annealing temperatures between 300 and 500 °C were applied to get the optimized electrode.

The morphologies of the as‐prepared samples were characterized by scanning electron microscopy (SEM). Figure S1, Supporting Information, shows the typical SEM image of Ni foam/CMO before (Ni foam/CMO‐350) and after P‐doping obtained at 350 °C (Ni foam/CMP‐350). The active material showed nanosheet morphology with the size of hundreds of nanometers to micrometers which covered the Ni foam evenly. As can be seen from high magnification SEM images in Figure S1d, Supporting Information, the Ni foam/CMP‐350 showed petal‐like nanostructure with relatively high surface area. Figure S2, Supporting Information, showed the energy‐dispersive X‐ray spectroscopy (EDX) mapping images of Ni foam/CMP‐350, which indicates an even distribution of materials supported on the Ni foam.

**Figure 2:**
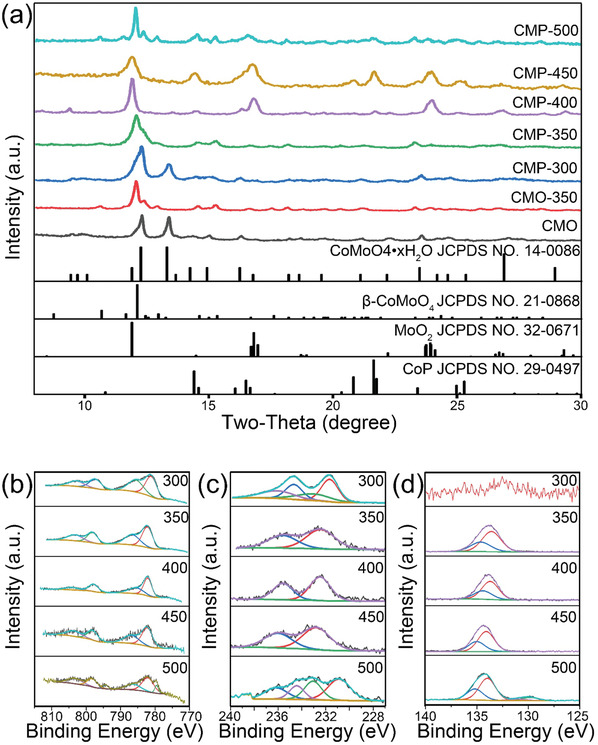
a) XRD patterns of CMO and CMP materials annealed at different temperatures. XPS spectra of b) Co, c) Mo, and d) P of Ni foam/CMP materials annealed at different temperatures (300, 350, 400, 450, and 500 °C).

X‐ray diffraction (XRD) and XPS were employed to characterize the structural and compositional features of the CMP material. **Figure** **2**a illustrates the XRD patterns of CoMoO_4_‐based powder samples. As can be seen, the CMO‐precursor obtained directly from hydrothermal process matched the structure of CoMoO_4_·*x*H_2_O (JCPDF No. 14–0086). The XRD pattern of CMP‐300 matched well with CoMoO_4_·*x*H_2_O, which indicate the structure did not change at the annealing temperature of 300 °C. When annealing at 350 °C for 1 h under N_2_ atmosphere, the structure changes from CoMoO_4_·*x*H_2_O to *β*‐CoMoO_4_ (JCPDF card No. 21–0868). *β*‐CoMoO_4_ exhibits a monoclinic structure with space group C2/m, in which Co^2+^ and Mo^6+^ cations are coordinated with six and four oxygen atoms in octahedral and tetrahedral environments respectively. If NaH_2_PO_2_ is introduced during the annealing process, P‐doped CoMoO_4_ displays the same pattern as CoMoO_4_ at 350 °C with a slight shift, suggesting the reaction between PH_3_ and *β*‐CoMoO_4_ does not yield any new product at this temperature. However, if the annealing temperature is raised above 400 °C, new peaks appear at 2*θ* between 15° and 25°. This indicates the phase separation of CoMoO_4_ above 400 °C to form MoO_2_ and cobalt oxide which could further react with PH_3_ and form CoP (JCPDF card No. 29–0497). The XRD peaks of CMO‐350 at 12.1°, 15.3°, and 23.3° could be indexed to (002), ($\bar{2}$22), and ($\bar{2}$04) planes of *β*‐CoMoO_4_ (JCPDF card No. 21–0868). The XRD peaks of CMP‐400 at 14.4° and 21.6° could be indexed to (011) and (211) planes of CoP (JCPDF card No. 29–0497). The XRD peaks of CMP‐400 at 16.8° and 23.9° could be indexed to ($\bar{2}$11) and ($\bar{3}$12) planes of MoO_2_ (JCPDF card No. 32–0671).

Typical XPS characterization was used to identify the elemental composition of the CMP samples. Figure 2b–d shows the high resolution XPS spectra of Co 2p, Mo 3d, and P 2p for Ni foam/CMP materials calcined at different temperatures. In the Co 2p region, Ni foam/CMP annealed from 300 to 450 °C showed two main peaks at the binding energies of 782.2 and 798.2 eV respectively, which were assigned to Co^2+^ species. Two peaks at binding energies of 786.7 and 803.1 eV were ascribed to satellite peaks of Co^2+^. For Ni foam/CMP‐500, two new peaks at the binding energy of 779.7 and 794.4 eV could be indexed to Co^3+^ species.^[^ ^28^ ^]^ Because of the strong P—O bond, cleavage of P—O, and formation of metal—phosphorus bonds can only occur at high temperature (above 773 K).^[^ ^29^ ^]^ The binding energies at 235.1 and 231.8 eV are assigned to Mo^6+^ 3d3/2 and 3d5/2, respectively.^[^ ^30^ ^]^ The high‐resolution P 2p spectrum showed the binding energies at 133.9 and 134.5 eV which were assigned to P^5+^ 2p1/2 and 2p3/2 respectively, except for Ni foam/CMP‐300 and Ni foam/CMP‐500. The P^5+^ could be derived from the oxidation of P and exist as PO_4_^3−^ group on the surface of the material. For Ni foam/CMP‐300, there was no obvious P signal appearing, which indicates P doping is unlikely to occur at the low annealing temperature of 300 °C. Upon calcination at 500 °C, two new peaks at 129.8 and 130.9 eV could be indexed to Co—P species.^[^ ^31^, ^32^ ^]^ The simultaneous occurrence of P^3−^ signals in the P XPS and CoP in the XRD patterns, suggests that upon calcination at 500 °C, P‐doped CoMoO_4_ partially decomposes to form CoP.^[^ ^33^ ^]^ Quantitative XPS analysis showed that the ratio of Co:Mo was 1:2.25 for Ni foam/CMO‐350 (Figure S3, Supporting Information) and the ratio of Co:Mo:P was 1:0.49:3.66 for Ni foam/CMP‐350. The reduction of Mo content suggests that upon P doping the MoO_4_^2−^ group is partially replaced by PO_4_^3−^. The characterization of surface PO_4_^3−^ groups is discussed in further detail using experimental IR spectra and computational results.

**Figure 3:**
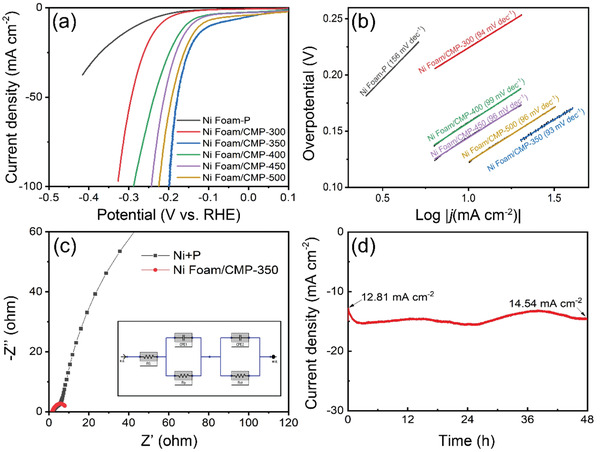
Electrocatalytic measurements of different electrodes for hydrogen evolution in 1 m KOH. a) The polarization curves of different samples. b) Tafel plots derived from the curves in (a). c) Nyquist plots of electrochemical impedance spectra (EIS) of Ni foam with P doping and Ni foam/CMP‐350 recorded in 1 m KOH solution. Inset: (c): Two‐time‐constant model equivalent circuit used for data fitting of EIS spectra. d) Chronoamperometric curve obtained with the Ni foam/CMP‐350 electrode.

The HER electrocatalytic performance of the Ni foam‐P, Ni foam/CMO‐350, Ni foam/CMP‐300, Ni foam/CMP‐350, Ni foam/CMP‐400, Ni foam/CMP‐450, and Ni foam/CMP‐500 were examined in 1 m KOH electrolyte. **Figure** **3**a and Figure S4, Supporting Information, present the relevant polarization curves. At a geometric current density of 10 mA cm^−2^, the overpotential for Ni foam/CMP‐350 is 94 mV, which is better than that of Ni foam‐P (280 mV), Ni foam/CMO‐350 (268 mV), Ni foam/CMP‐300 (224 mV), Ni foam/CMP‐400 (158 mV), Ni foam/CMP‐450 (145 mV), and Ni foam/CMP‐500 (121 mV). To quantify the HER activity of the Ni foam, we performed catalytic activity tests upon a Ni foam sample after it underwent the same phosphating process as the CMO samples. The Ni foam exhibits negligible HER activity. To obtain the kinetic information of the as‐prepared electrodes, the corresponding Tafel plots are given in Figure 3b. The Ni foam/CMP‐350 exhibited a Tafel slope of 94 mV dec^−1^, which suggests in addition to Heyrovsky reaction, the Volmer reaction is also the rate‐determining step.^[^ ^34^ ^]^ Figure 3c illustrates the electrochemical impedance spectroscopy (EIS) spectra of the catalysts: *R*_s_ represents the overall series resistance; CPE1 and CPE2 represent the constant phase element and resistance related to surface porosity; *R*_p_ and *R*_ct_ represent the charge transfer resistance related to the HER process. The excellent catalytic performance of Ni foam/CMP‐350 is mirrored from its small *R*_ct_ value determined via the measurements (Figure 3c), which shows the electrical conductivity is higher and the charge‐transfer capability is improved.^[^ ^13^, ^35^ ^]^ Furthermore, a stability test was performed on Ni foam/CMP‐350 at a fixed potential of −150 mV versus RHE, the current density of Ni foam/CMP‐350 increased by 13% from 12.81 to 14.54 mA cm^−2^ after 48 h. An activation period could be witnessed for 2 h. The current density was increased to 15.27 mA cm^−2^. After that, the curve was relatively stable and slightly decreased to 14.54 mA cm^−2^ in the end, which indicates excellent stability of the catalyst. The ratio of the refilling P was optimized by the change of annealing time and the amount of NaH_2_PO_2_·H_2_O applied. Table S1, Supporting Information, showed the XPS quantitative results of as‐prepared electrodes and Figure S4, Supporting Information, showed HER results of electrodes prepared at different conditions. The results indicated the reaction time did not influence too much on the ratio of the refilling of P atoms, while the amount of NaH_2_PO_2_·H_2_O would do. The results showed in Table S1, Supporting Information, indicates with increasing amount of NaH_2_PO_2_·H_2_O, the refilling of P atoms would increase in the electrodes. Ni foam/CMP‐500 mg and Ni foam/CMP‐700 mg showed similar performance while the Ni foam/CMP‐50 mg and Ni foam/CMP‐200 mg showed a relatively low performance. These experimental results proved that the refilling of P atoms is correlated with catalytic activity at a low refilling of P. The Ni foam/CMP‐550 showed a lower performance than Ni foam/CMP‐350. Figure S5, Supporting Information, shows the XPS spectra of Ni foam/CMP‐350 before and after the stability test. The XPS spectra of Co slightly shifts to lower binding energy after stability test. The catalyst surface undergoes a reconstruction process as the HER proceeds. The catalysts surface would change from P‐rich to Co‐rich. This phenomenon is understood as a result of hydroxide‐replacing‐polyphosphate process. The resulting reduced surfaces can be assigned to Co_2_P‐like species.^[^ ^36^, ^37^ ^]^ In the P 2p spectrum, P—O component shifts from 134.2 to 132.9 eV, which could be assigned to phosphite or hypophosphite residue present on the catalyst surface under HER condition.^[^ ^37^ ^]^ Figure S6, Supporting Information, shows the TEM images of Ni foam/CMP‐350 before (Figure S6a, Supporting Information) and after (Figure S6b, Supporting Information) the chronoamperometry test at a 100 mV overpotential for 24 h. The planes of *β*‐CoMoO_4_ could be found on both images and the structure of the catalyst did not change during the stability test, which confirmed the remarkable stability of the electrode. To better understand the remarkable catalytic property of Ni foam/CMP‐350, the electrochemically active surface area was investigated using a typical cyclic voltammetry (CV) method (Figure S7, Supporting Information). The double‐layer capacitance (*C*_dl_) of Ni foam/CMP‐350 is 35.5 mF cm^−2^ while the *C*_dl_ for Ni foam/CMP‐500 is 42.1 mF cm^−2^. The excellent HER performance of Ni foam/CMP‐350 is further shown due to its higher current density at a lower potential and relatively low *C*_dl_ value compared to Ni foam/CMP‐500. The *C*_dl_ of Ni foam/CMP‐300, Ni foam/CMP‐400, and Ni foam/CMP‐450 are around 20 mF cm^−2^ (Figure S8, Supporting Information). A full water splitting test was performed in a two‐electrode cell in 1 m KOH at a scan rate of 2 mV s^−1^ (Figure S9, Supporting Information). The onset potential for overall water splitting was relatively low when compared to other electrocatalysts, which indicated that Ni foam/CMP‐350 is also active for the oxygen evolution reaction. Figure S10, Supporting Information, showed the EIS results of different electrodes recorded in 1 m KOH solution under open circuit condition. The resistance values of P‐doped Ni foam, Ni foam/CMP‐300, Ni foam/CMP‐350, Ni foam/CMP‐400, Ni foam/CMP‐450, and Ni foam/CMP‐500 are 2.46, 1.16, 1.58, 1.95, 1.55, and 1.49 Ω, respectively, which indicates the resistance would be lower with P‐doped CoMoO_4_ supported on the Ni foam compared with P‐doped Ni foam. And all Ni foam/CMP electrodes showed a small value of resistance which indicated a fast electron transfer ability. The Faraday efficiency (FE) experiment was conducted, and the result showed a nearly 100% FE for hydrogen evolution (Figure S11, Supporting Information).

The defect chemistry of P‐CoMoO_4_, was characterized using hybrid exchange density functional theory calculations. Following indications from XPS that P is in the 5+ oxidation state in the active catalyst, we studied its incorporation of P as a molecular phosphate anion (PO_4_^3−^). The structural and chemical similarity of these molecular ions strongly implies that P^5+^ replacing Mo^6+^ as a polyanion is the most likely dopant configuration. It has been suggested that P can replace surface O^2−^ in its 3− oxidation state,^[^ ^27^ ^]^ however this appears unlikely when P is in its 5+ oxidation state as in the current Ni foam/CMP‐350 catalyst. P doping in bulk *β*‐CMO was accomplished in our calculations by incorporating 1 P atom in a 1 × 1 × 2 supercell comprising of 48 atoms. Indeed this PO_4_^3−^/MoO_4_^2‐^ replacement in bulk is found to cause only a localized structural rearrangement, corresponding to a contraction of the M—O bond lengths from 1.73–1.79 Å in the MoO_4_^2‐^ unit to 1.59–1.67 Å for the PO_4_^3−^. The geometry optimized cell volume decreases by 2.8% upon doping, despite the high P content (12.5% of the Mo sites) in the computational cell. A comparison of experimental and simulated lattice parameters are shown in Table S2, Supporting Information, highlighting the ease of incorporation and small local distortion caused by P‐doping on the Mo site.

Replacement of Mo^6+^ by P^5+^ generates a charge imbalance, which surprisingly in all our calculations is compensated by an electron hole localized on one of the PO_4_^3−^ oxygen ions rather than the oxidation of Co^2+^ to Co^3+^. Doping generates an acceptor state in the band gap of CMO that will enhance electronic conductivity and is able to accommodate additional electrons at negative applied potential, both beneficial for the HER. The electron hole defect has been also examined at the surface of *β*‐CoMoO_4._ Structural analysis indicates that the (110) plane has the lowest density of inter‐plane bonds and thus represents a preferential cleavage plane. In this direction adjacent, (110) planes are connected by oxygen ions bridging between one MoO_4_ and one CoO_6_ polyhedra in a near perpendicular direction. The different bond lengths of 1.73–1.79 Å for Mo—O and 2.03–2.13 Å for Co—O indicates that the Co—O bond is the easiest to cleave. In addition, the Co—O bond affected is the longest in the CoO_6_ octahedron. Cleavage is obtained by leaving each of these oxygens attached to the MoO_4_^2−^ unit it belongs to, which therefore leaves an exposed 5 coordinated Co^2+^ ion. This corresponds to a $\sqrt{2}\;\times \;\sqrt{2}$ reconstruction of the surface. The (110) surface energy of pure CoMoO_4_ is calculated to be only 0.32 J m^−1^ indicating indeed a stable and easily cleaved surface.

**Figure 4:**
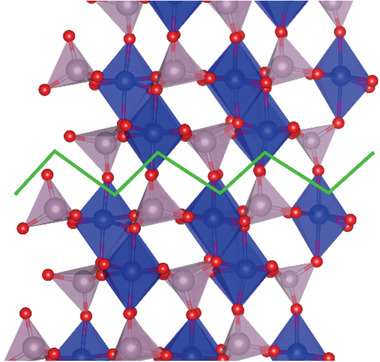
Bulk CoMoO_4_ and the (110) cleavage plane. O is shown by red spheres, Co by blue, Mo by mauve, and the cleavage plane by the green line. In the surface Co and Mo retain their octahedral and tetrahedral environment respectively. The surface terminates with Co and O with the latter protruding out from the surface.

The (110) surface in our calculations was generated by orientating atoms along the direction perpendicular to the surface and cleaving a slab ≈18Å thick. A $\sqrt{2}\;\times \;\sqrt{2}$ surface reconstruction yields a cell with two surface Mo ions of which one was replaced by P on both upper and lower surfaces of the slab. The surface cleavage is shown in **Figure** **4**, where we observe the under‐coordinated CoO_5_ surface groups and the MoO_4_ tetrahedra, the latter with one O protruding from the surface.

**Figure 5:**
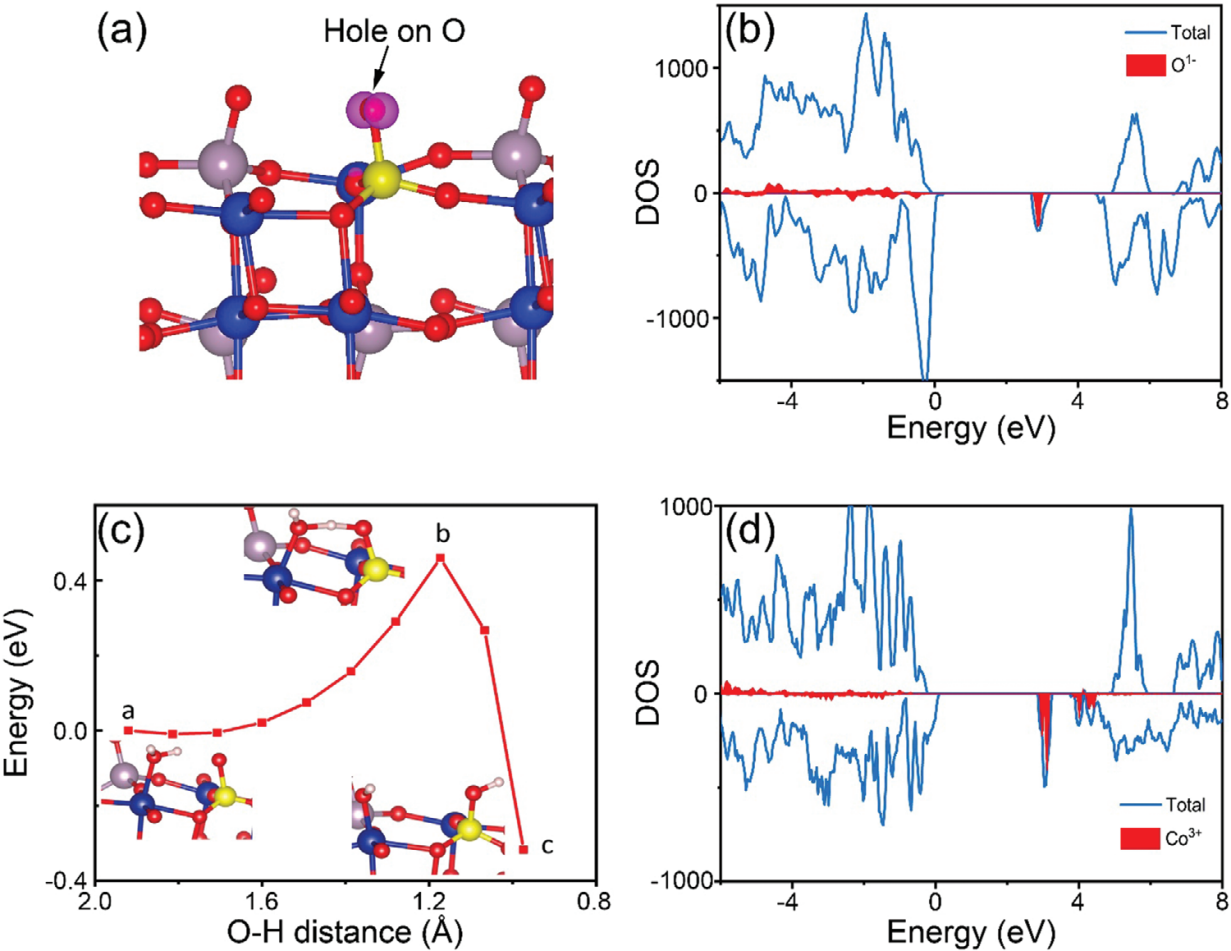
a) Spin density map showing a localized electron hole on a surface oxygen, created upon doping. P occupies a Mo site and is shown by yellow spheres. b) DOS showing the accepter level within the bandgap corresponding to O^1−^. c) The energy barrier for the dissociation of water on the CMP surface to form Co—OH surface species in the absence of an external potential. Points (A), (B), and (C) represent the initial transition and final geometries for the section. d) DOS showing an accepter level within the bandgap corresponding to Co^3+^. An electron from the external circuit will localize on Co creating Co^2+^ and OH^−^ will likely desorb.

Replacement of a surface MoO_4_^2−^ unit with PO_4_^3−^ yields a similar O^−1^ acceptor defect state to the bulk, the hole is localized on the phosphate oxygen ion directly exposed on the surface. This electronic state is shown by its spin density plot in **Figure** **5**a. Substitution of MoO_4_^2−^ by PO_4_^3−^ at the (110) surface is calculated to be 0.12 eV more stable than in the bulk; therefore, our calculations support an enhanced concentration of P ions at the surface and a high hetero‐catalytic effect of P doping in *β*‐CoMoO_4_ in agreement with the quantitative XPS analysis_._ The density of states (DOS) for the surface (Figure 5b) clearly shows the acceptor state within the band gap associated to O^1−^. The exposed O^1−^ species is expected to show a high chemical activity.

The high reactivity of the surface O^1−^ ion is confirmed by its ability to induce the homolytic dissociation of a water molecule: the H∙ moiety binds to the O^1−^ reducing it and forming a P—OH group, while the hydroxyl radical ∙OH is adsorbed onto the adjacent five‐coordinated Co^2+^ ion, eventually yielding a Co^3+^—OH^−^ surface species. Calculations show that this surface passivation mechanism has a low barrier of 0.46 eV and is exothermic by 0.32 eV (Figure 5c) and confirms the high reactivity of the doped surface. This barrier will be lowered by application of a negative potential that populates the acceptor state at the surface and thus favors the reduction of water. The passivation of the surface yields an enhanced concentration of OH groups on the P‐doped surface of CoMoO_4_. The DOS (Figure 5d) for the passivated surface still shows an acceptor state in the band gap, this time associated with the oxidized Co^3+^ ion. Application of a negative potential during the electrocatalytic cycle is expected to induce desorption of the hydroxide ion from the surface, combined with the localization of electrons on the surface‐exposed acceptor state, suggesting enhanced HER activity. Although the exact mechanism of H_2_ evolution is still not explicitly understood, reduction of water from either the P—O^1‐^ or the passivated Co^3+^—OH^−^ surface sites is calculated to be an exothermic process consistent with the high activity measured experimentally.

**Figure 6:**
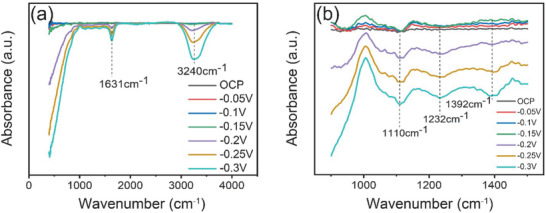
a) In situ ATR‐IR spectra of CMP‐350 materials in deoxygenated 0.1 m KOH solution at different applied voltage for 10 min. b) Enlarged graph from (a). The potential has converted to V versus RHE.

To further study the P‐doping effect and catalytic mechanism as well as verify the computational results, in situ IR spectroscopy was employed to probe the change of CMP‐350 as a function of applied potential. The IR spectra was obtained after the applied voltage was kept for 10 min. The peaks at 1631 and 3240 cm^−1^ could be related to O—H bending and O—H stretching vibrations respectively (**Figure** **6**a). This result indicates an enhanced concentration of H‐containing groups on the active surface in the absence of applied potential, consistent with the composition of the active surface (P—OH, Co^3+^—OH^−^) discussed in the computational work. Upon application of a negative potential the OH concentration on the surface decreases, indicating that desorption of surface ions OH^−^ ions upon occupation of the acceptor defect state inferred theoretically does indeed take place. With increasing negative potential O—H groups expelled from the interface between the electrode and the electrolyte. This implies that more of the O—H groups passivating the surface are destabilized and expelled, which may further facilitate the Volmer step corresponding to the dissociation of water (H_2_O + e^−^ →H* + OH^−^).^[^ ^38^ ^]^ Thus, the decrease in concentration of OH^−^ might lead to a better HER performance. Figure 6b shows the enlarged area from Figure 6a. The peaks at 1110 and 1232 cm^−1^ can be assigned to the P—O stretching modes with oxygen bridging to a second metal ion, hence to P—O—Co groups. The higher frequency mode at 1392 cm^−1^ is not usually observed in phosphate minerals, but is present in phosphate glasses, where it is assigned to the stretching of terminal P=O groups. This analysis indicates that P indeed exists as a phosphate group, in agreement with XPS results, but also that it is under‐coordinated such as would be the case for surface exposed PO_4_^3−^ groups identified in the computational work.^[^ ^39^ ^]^ In addition, the fact that the P=O contribution changes as a function of the applied voltage confirms it is active in the HER mechanism. These results suggest that the replacement of P to Mo is beneficial to the HER process by creating highly reactive surface active sites.

## 3 Conclusions

In this work, a series of P‐doped CoMoO_4_ nanostructures on Ni foam were successfully synthesized by facile hydrothermal‐annealing. Quantitative XPS analysis showed a reduced content of Mo after P doping, which means that some of the surface MoO_4_^4−^ units are partially replaced by PO_4_^3−^ units. To further understand the P‐doping effect and catalytic mechanism, a combined in situ IR spectroscopy and computational study were employed to uncover the active species of the best performing sample. The results indicate the presence of surface O—H groups whose concentration decreases at lower potentials. Electron holes localize on exposed surface O species, which can induce the homolytic dissociation of water creating an O—H passivated surface with Co^3+^—OH^−^ sites, which are the active sites for the HER.

## 4 Experimental Section

##### Material

Sodium hypophosphite monohydrate (NaH_2_PO_2_·H_2_O) was purchased from Sigma‐Aldrich (UK) Co., Ltd. Sodium molybdate dihydrate (Na_2_MoO_4_·2H_2_O) was purchased from Merck (USA) Co., Ltd. Cobalt nitrate hexahydrate (Co(NO_3_)_2_·6H_2_O) was purchased from Alfa Aesar (UK) Co., Ltd. All chemicals were used as received without further purification.

##### Preparation of Cobalt Molybdenum Oxide (CoMoO_4_) Precursor and Ni Foam/CoMoO_4_ Precursor

In a typical experimental process, 0.384 g of Co(NO_3_)_2_·6H_2_O and 0.272 g of Na_2_MoO_4_·2H_2_O were mixed in 12 mL deionized water to generate a light purple solution. The mixed solution was stirred for about 30 min. Commercial Ni foams (5 cm × 1 cm) were used as the substrates for free‐standing electrodes. Ni foam was put into 0.5 m hydrochloric acid solution for ≈5 min under ultrasonication to remove the possible surface oxide layers. Afterward, the Ni foam was washed by DI water and dried in an oven at 60 °C overnight. 20 mL hydrothermal reactors were used and Ni foams were placed against the side wall. Then, the as‐prepared solutions were transferred to the reactors. The reactors were tightened and kept in the furnace at 120 °C for 12 h and left to cool down to room temperature naturally. After reaction, the final Ni foam/CoMoO_4_‐presursor were immersed in ultrasonic vibration for 5 s to remove the loosely attached materials. Then the Ni foam/CoMoO_4_‐presursor was washed with deionized water and absolute ethanol four times and dried in a vacuum oven at 110 °C for 5 h. In order to obtain the powder samples for XRD analysis, the samples were collected through centrifugation and washed by deionized water and absolute ethanol four times. The purple powder could be obtained after freeze‐drying for 24 h to get the CoMoO_4_‐precursor.

##### Preparation of Ni Foam/CoMoO_4_ with Phosphorous Doping and CoMoO_4_ Powder with Phosphorous Doping

The Ni foam/CoMoO_4_‐precursor was heated in a tube furnace at 300 °C for 1 h with a heating rate of 20 °C min^−1^ under N_2_ atmosphere to improve the crystallinity. Then 500 mg of NaH_2_PO_2_·H_2_O was pushed into the tube furnace to heat for one hour under N_2_ atmosphere. The waste gas was absorbed by the sodium hypochlorite solution. The final product was taken out when the tube furnace cooled down to room temperature. Different heating temperature (350, 400, 450, 500, and 550 °C) were applied to investigate the influence of annealing temperature while other conditions were kept the same. The samples obtained from different temperatures were marked as Ni foam/CMP‐350, Ni foam/CMP‐400, Ni foam/CMP‐450, Ni foam/CMP‐500, and Ni foam/CMP‐550, separately. Different amount of NaH_2_PO_2_·H_2_O (50, 200, and 700 mg) were applied to investigate the influence of P source while other conditions were the same with Ni foam/CMP‐350. The samples obtained were marked as Ni foam/CMP‐50 mg, Ni foam/CMP‐200 mg, and Ni foam/CMP‐700 mg. Different annealing time (0.5 h and 2 h) with NaH_2_PO_2_·H_2_O was applied to investigate the influence of annealing time while other conditions were the same with Ni foam/CMP‐350. The samples obtained from different annealing time were marked as Ni foam/CMP‐0.5 h and Ni foam/CMP‐2 h. To make a comparison, the Ni foam was put into a quartz crucible and heated in a tube furnace at 350 °C for 1 h under N_2_ atmosphere with a heating rate of 20 °C min^−1^ and marked as Ni foam‐P and a pure Ni foam was marked as pure Ni. The as‐prepared CoMoO_4_‐precursor powder samples were treated as the same process as Ni foam/CoMoO_4_ and the final products were marked as CMO‐350, CMP‐300, CMP‐350, CMP‐400, CMP‐450, and CMP‐500, respectively.

##### Characterization

The morphology and microstructures of samples were characterized by scanning electron microscope (Carl Zeiss EVO MA10) and transmission electron microscope (JEOL, JEM‐2100). The phase and chemical composites were recorded by a STOE SEIFERT diffractometer (Mo source radiation), XPS (Thermo scientific K‐alpha photoelectron spectrometer). The mass of the electrodes was weighed accurately by an analytical balance (Ohaus; *δ* = 0.01 mg).

##### Electrochemical Tests

Electrochemical measurements of the as‐prepared materials on Ni foam were conducted in a three‐electrode cell. 1 m KOH solution was prepared as the electrolyte. A graphite rod was used as the counter electrode and an Ag/AgCl (3 m KCl) electrode was used as a reference electrode. The as‐prepared Ni foam samples were used as the working electrode. The self‐standing electrodes were connected directly with an area of ≈1 cm^2^ immersed in electrolyte for testing. The cyclic voltammetry (CV) and linear sweep voltammetry (LSV) measurements were carried out by a Gamry interface 1000 potentiostat. Polarization data were collected at a scan rate of 5 mV s^−1^. All the potentials were measured against an Ag/AgCl electrode and were converted into the potential versus the reversible hydrogen electrode (RHE) according to *E*_RHE_ = *E*_Ag/AgCl_ + 0.197 + 0.059 pH. Tafel slopes were determined by fitting the linear regions of the Tafel plots according to the Tafel equation (*η* = *b* log (*j*) + *a*) by replotting the polarization curves. The long‐term stability was evaluated by the chronoamperometry measurement. EIS was performed with frequencies from 0.1 to 100 000 Hz with an amplitude of 10 mV. All the LSV measurements were presented with *iR* compensation.

##### Computational Details

Density functional theory calculations were performed using the CRYSTAL17 software package, which treated crystalline orbitals as a linear combination of atomic orbitals, represented by a linear combination of Gaussian functions. All electron basis sets (BS) of at least double zeta plus polarization quality were used for H, P, O, Co, and a small core pseudopotential for Mo. The BS were obtained from the CRYSTAL online library (www.crystal.unito.it/basis-sets.php) with codes: H_pob_TZVP_2012; P_85‐21d1G_zicovich_2002 O_8‐411d1_cora_2005; Co_double_zeta_ruiz_2003; Mo_POB_DZVP_2018. The PBE0 hybrid exchange functional was used to approximate the electron exchange and correlation. All truncation and convergence criteria were set to default values as specified by the code for the geometry optimizations of both bulk and surface calculations. Reciprocal space was sampled using an 8 × 8 × 8 Monkhorst‐Pack grid giving 150 k points in the irreducible Brillouin zone of bulk CoMoO_4_. The reciprocal space sampling was reduced to 4 × 4 × 4 in the supercells used to study the P‐doped systems.

P doping was accomplished by incorporating 1 P atom in a 1 × 1 × 2 supercell in bulk CoMoO_4_ comprising of 48 atoms. The (110) surface was generated by orienting atoms along the direction perpendicular to the surface and cleaving a slab ≈18 Å thick comprised of 48 atoms. A $\sqrt{2}\;\times \;\sqrt{2}$ surface reconstruction yielded a cell with two surface Mo ions, of which one was replaced by P. Reaction barriers were calculated using the distinguished reaction coordinate method, where a distance (O—H) represented the reaction coordinate. This distance was held constant during sequential partial geometry optimizations extrapolated along the reaction coordinate to calculate the total energy relative to the initial state. In this instance, an extrapolation of 10 points which corresponded to a step of ≈0.1 Å was chosen.

##### In Situ ATR‐IR Characterization

A Bruker Tensor 27 IR spectrometer with a diamond crystal single‐reflection internal reflection element ATR prism accessory was used for all experiments. The instrument was fitted with a room temperature DLaTGS detector at 4 cm^−1^ resolution. The counter electrode (CE) was a Pt wire and the reference electrode (RE) was Ag/AgCl filled with saturated KCl solution. The potential was controlled with a Palmsens Emstat2 potentiostat (Palmsens, NL). The electrode was equilibrated in the 0.1 m KOH electrolyte (deoxygenated for 20 min prior with Ar) for ≈10 min and an IR background spectrum was obtained at open circuit potential. After which, the potential was applied, and spectra were recorded relative to the spectrum of the equilibrated sample. Applied potentials were adjusted from 0 to −0.3 V versus RHE with a step of −0.05 V. Each spectrum was recorded after keeping the specific potential for 10 min.

##### Determination of Faradaic Efficiency

The generated gas was confirmed by calibrated Mass Spectrometer (Hiden Analytical QGA) analysis and measured quantitatively using a mass flow meter with a fixed flow rate of 20 sccm of N_2_. An H‐cell was used to perform HER test with a fixed current density of 10 mA cm^−2^. The FE test was stabilized for 10 min before collecting the data. The FE was calculated by comparing the amount of measured hydrogen generated by potentialstatic cathodic electrolysis with calculated hydrogen (assuming 100% FE). The rough agreement of both values suggested nearly 100% FE for hydrogen evolution.

## Conflict of Interest

The authors declare no conflict of interest.

## Supporting information

Supporting Information

## References

[1] Z. W. She , J. Kibsgaard , C. F. Dickens , I. Chorkendorff , J. K. Nørskov , T. F. Jaramillo , Science 2017, 355, eaad4998. 28082532 10.1126/science.aad4998

[2] Y. Y. Ma , Z. L. Lang , L. K. Yan , Y. H. Wang , H. Q. Tan , K. Feng , Y. J. Xia , J. Zhong , Y. Liu , Z. H. Kang , Y. G. Li , Energy Environ. Sci. 2018, 11, 2114.

[3] D. Merki , X. Hu , Energy Environ. Sci. 2011, 4, 3878.

[4] L. Ji , C. Lv , Z. Chen , Z. Huang , C. Zhang , Adv. Mater. 2018, 30, 1705653. 10.1002/adma.201705653 29333739

[5] Y. R. Zheng , P. Wu , M. R. Gao , X. L. Zhang , F. Y. Gao , H. X. Ju , R. Wu , Q. Gao , R. You , W. X. Huang , S. J. Liu , S. W. Hu , J. Zhu , Z. Li , S. H. Yu , Nat. Commun. 2018, 9, 2533. 29955067 10.1038/s41467-018-04954-7 PMC6023930

[6] R. Gusmão , Z. Sofer , M. Pumera , Adv. Funct. Mater. 2019, 29, 1805975.

[7] C. L. Bentley , C. Andronescu , M. Smialkowski , M. Kang , T. Tarnev , B. Marler , P. R. Unwin , U. P. Apfel , W. Schuhmann , Angew. Chem., Int. Ed. 2018, 57, 4093. 10.1002/anie.201712679 29377499

[8] H. Xu , J. Wan , H. Zhang , L. Fang , L. Liu , Z. Huang , J. Li , X. Gu , Y. Wang , Adv. Energy Mater. 2018, 8, 1800575.

[9] A. G. Vidales , S. Omanovic , Electrochim. Acta 2018, 262, 115.

[10] Y. Huang , J. Ge , J. Hu , J. Zhang , J. Hao , Y. Wei , Adv. Energy Mater. 2018, 8, 1701601.

[11] W. Lei , Y. Mi , R. Feng , P. Liu , S. Hu , J. Yu , X. Liu , J. A. Rodriguez , J. Wang , L. Zheng , K. Tang , S. Zhu , G. Liu , M. Liu , Nano Energy 2018, 50, 552.

[12] C. Wang , T. Wang , J. Liu , Y. Zhou , D. Yu , J. K. Cheng , F. Han , Q. Li , J. Chen , Y. Huang , Energy Environ. Sci. 2018, 11, 2467.

[13] Y. Y. Ma , C. X. Wu , X. J. Feng , H. Q. Tan , L. K. Yan , Y. Liu , Z. H. Kang , E. B. Wang , Y. G. Li , Energy Environ. Sci. 2017, 10, 788.

[14] Y. Zheng , Y. Jiao , A. Vasileff , S. Qiao , Angew. Chem., Int. Ed. 2018, 57, 7568. 10.1002/anie.201710556 29194903

[15] Y. Li , H. Wang , L. Xie , Y. Liang , G. Hong , H. Dai , J. Am. Chem. Soc. 2011, 133, 7296. 21510646 10.1021/ja201269b

[16] Y. Ouyang , Q. Li , L. Shi , C. Ling , J. Wang , J. Mater. Chem. A 2018, 6, 2289.

[17] P. Niu , M. Qiao , Y. Li , L. Huang , T. Zhai , Nano Energy 2018, 44, 73.

[18] Z. Fang , L. Peng , Y. Qian , X. Zhang , Y. Xie , J. J. Cha , G. Yu , J. Am. Chem. Soc. 2018, 140, 5241. 29608305 10.1021/jacs.8b01548

[19] Z. Y. Yu , C. C. Lang , M. R. Gao , Y. Chen , Q. Q. Fu , Y. Duan , S. H. Yu , Energy Environ. Sci. 2018, 11, 1890.

[20] R. Li , D. Zhou , J. Luo , W. Xu , J. Li , S. Li , P. Cheng , D. Yuan , J. Power Sources 2017, 341, 250.

[21] Q. Liu , J. Tian , W. Cui , P. Jiang , N. Cheng , A. M. Asiri , X. Sun , Angew. Chem., Int. Ed. 2014, 53, 6710. 10.1002/anie.201404161 24845625

[22] W. Cui , Q. Liu , Z. Xing , A. M. Asiri , K. A. Alamry , X. Sun , Appl. Catal., B 2015, 164, 144.

[23] C. Wan , Y. N. Regmi , B. M. Leonard , Angew. Chem., Int. Ed. 2014, 53, 6407. 10.1002/anie.201402998 24827779

[24] H. Vrubel , X. Hu , Angew. Chem., Int. Ed. 2012, 51, 12703. 10.1002/anie.201207111 23143996

[25] G. He , X. Han , B. Moss , Z. Weng , S. Gadipelli , F. Lai , A. G. Kafizas , D. J. L. Brett , Z. X. Guo , H. Wang , I. P. Parkin , Energy Storage Mater. 2018, 15, 380.

[26] L. Yan , L. Cao , P. Dai , X. Gu , D. Liu , L. Li , Y. Wang , X. Zhao , Adv. Funct. Mater. 2017, 27, 1703455.

[27] S. Li , N. Yang , L. Liao , Y. Luo , S. Wang , F. Cao , W. Zhou , D. Huang , H. Chen , ACS Appl. Mater. Interfaces 2018, 10, 37038. 30285410 10.1021/acsami.8b13266

[28] Y. Lin , M. Liu , Y. Pan , J. Zhang , J. Mater. Sci. 2017, 52, 10406.

[29] P. Xiao , W. Chen , X. Wang , Adv. Energy Mater. 2015, 5, 1500985.

[30] G. Humagain , K. MacDougal , J. MacInnis , J. M. Lowe , R. H. Coridan , S. MacQuarrie , M. Dasog , Adv. Energy Mater. 2018, 8, 1801461.

[31] L. Yan , S. Zhao , Y. Li , B. Zhang , J. Zhu , Z. Liu , X. Yuan , J. Yu , H. Zhang , P. K. Shen , Mater. Today Energy 2019, 12, 443.

[32] H. Liu , D. Liu , M. Gu , Z. Zhao , D. Chen , P. Cui , L. Xu , J. Yang , Mater. Today Energy 2019, 14, 100336.

[33] D. Wang , X. Zhang , D. Zhang , Y. Shen , Z. Wu , Appl. Catal., A 2016, 511, 11.

[34] R. Xu , L. Kang , J. Knossalla , J. Mielby , Q. Wang , B. Wang , J. Feng , G. He , Y. Qin , J. Xie , A. C. Swertz , Q. He , S. Kegnæs , D. J. L. Brett , F. Schüth , F. R. Wang , ACS Nano 2019, 13, 2463. 30649849 10.1021/acsnano.8b09399

[35] D. Strmcnik , P. P. Lopes , B. Genorio , V. R. Stamenkovic , N. M. Markovic , Nano Energy 2016, 29, 29.

[36] Z. Wu , L. Huang , H. Liu , H. Wang , ACS Catal. 2019, 9, 2956.

[37] Z. Wu , Q. Gan , X. Li , Y. Zhong , H. Wang , J. Phys. Chem. C 2018, 122, 2848.

[38] F. Safizadeh , E. Ghali , G. Houlachi , Int. J. Hydrogen Energy 2015, 40, 256.

[39] V. N. Rai , B. N. R. Sekhar , P. Tiwari , R. J. Kshirsagar , S. K. Deb , J. Non‐Cryst. Solids 2011, 357, 3757.

