# Supplementary material for: The Role of Phosphate Group in Doped Cobalt Molybdate: Improved Electrocatalytic Hydrogen Evolution Performance
Source: Adv Sci (Weinh). 2020 Apr 22;7(12):1903674. doi: 10.1002/advs.201903674 (PMC7312439; doi:10.1002/advs.201903674)
Supplement: Supplementary file 1 — Supporting Information [file ADVS-7-1903674-s001.pdf]

Supporting Information

The Role of Phosphate Group in Doped Cobalt Molybdate: Improved Electrocatalytic Hydrogen Evolution Performance

Siyu Zhao[1], Jasper Berry-Gair[1], Wenyao Li[2,3], Guoqiang Guan[5], Manni Yang[1], Jianwei Li[1], Feili Lai[5], Furio Corà[1], Katherine Holt[1], Dan J.L. Brett[3], Guanjie He[1,3,4]* and Ivan P. Parkin[1]*

[1]*Christopher Ingold Laboratory, Department of Chemistry, University College London, 20 Gordon Street, London WC1H 0AJ, U.K. Email: g.he@ucl.ac.uk; i.p.parkin@ucl.ac.uk*

[2]*School of Materials Engineering, Shanghai University of Engineering Science, Shanghai 201620, China.*

[3]*Electrochemical Innovation Lab, Department Chemical Engineering, University College London, London WC1E 7JE, U.K.*

[4]*School of Chemistry, University of Lincoln, Joseph Banks Laboratories, Green Lane, Lincoln, LN6 7DL, United Kingdom*

[5]*State Key Laboratory for Modification of Chemical Fibers and Polymer Materials, College of Materials Science and Engineering, Donghua University, Shanghai 201620, China*

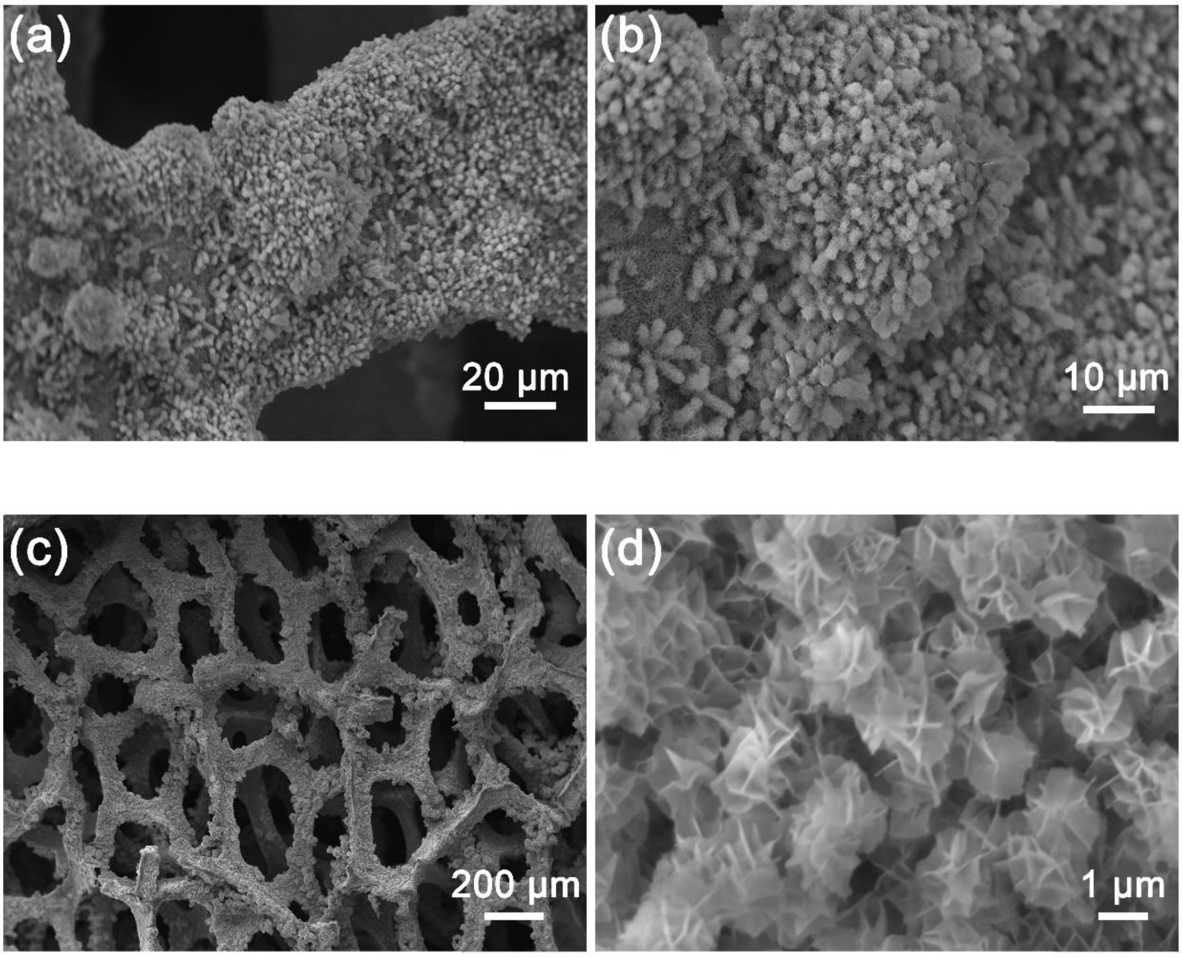

Figure S1. (a, b) SEM image of Ni Foam/CMO-350. (c, d) SEM image of Ni Foam/CMP-350.

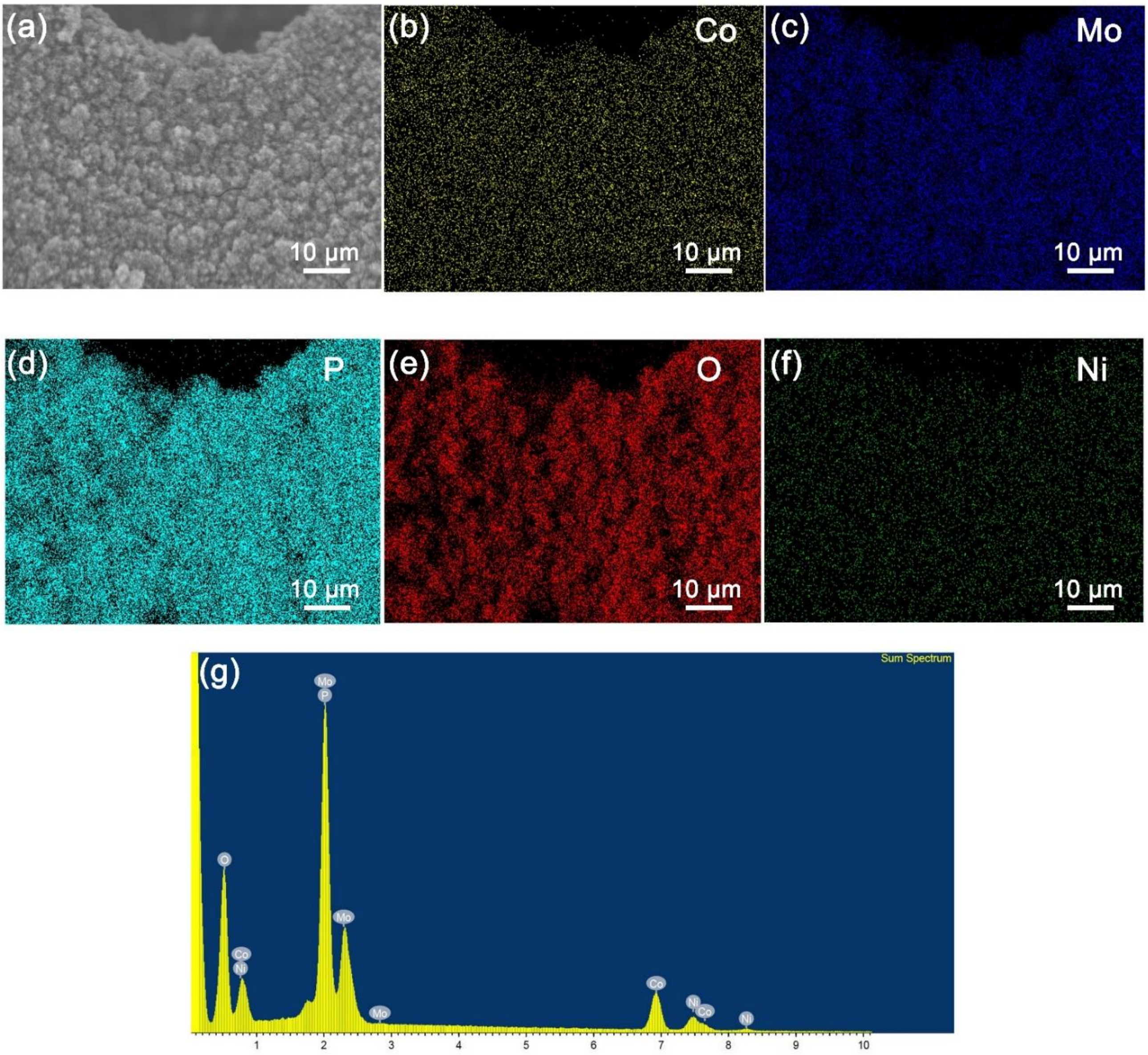

Figure S2. EDX mapping images of Ni foam/CMP-350.

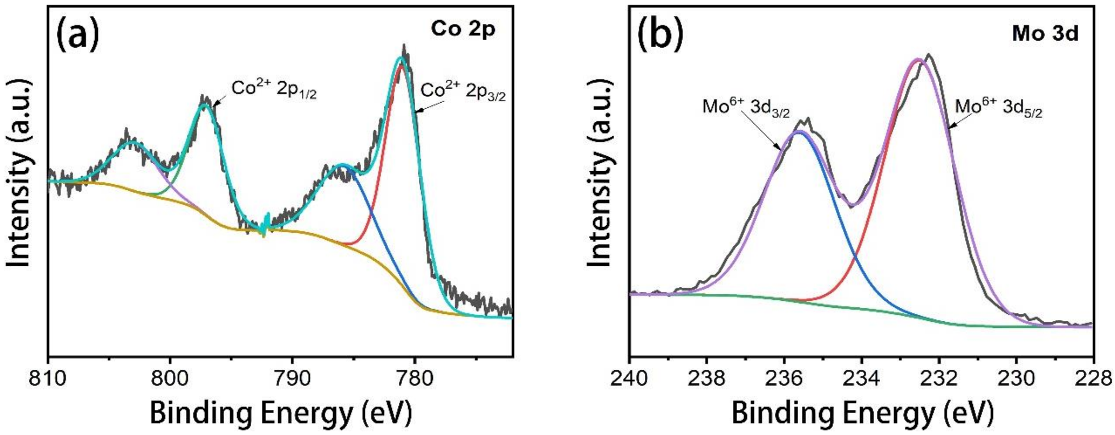

Figure S3. XPS spectra of (a) Co, (b)Mo of Ni Foam/CMO-350

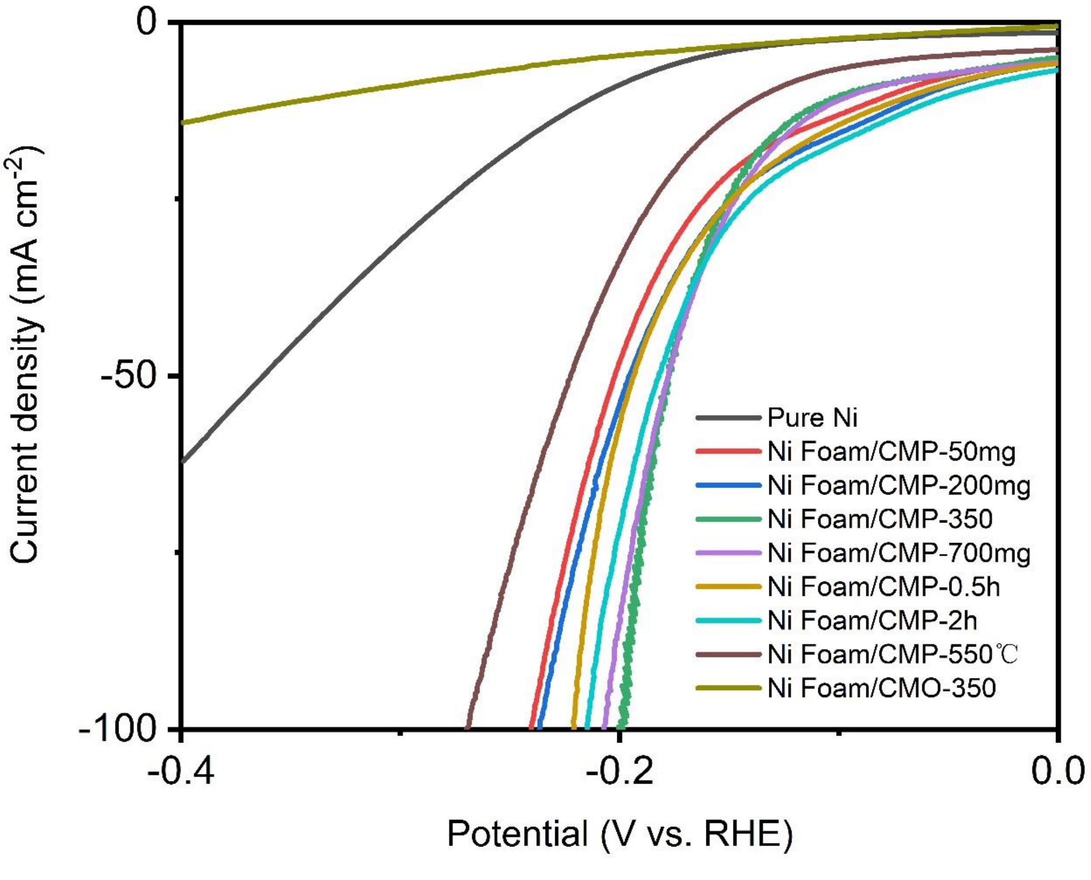

Figure S4. Polarization curves of electrodes in 1M KOH.

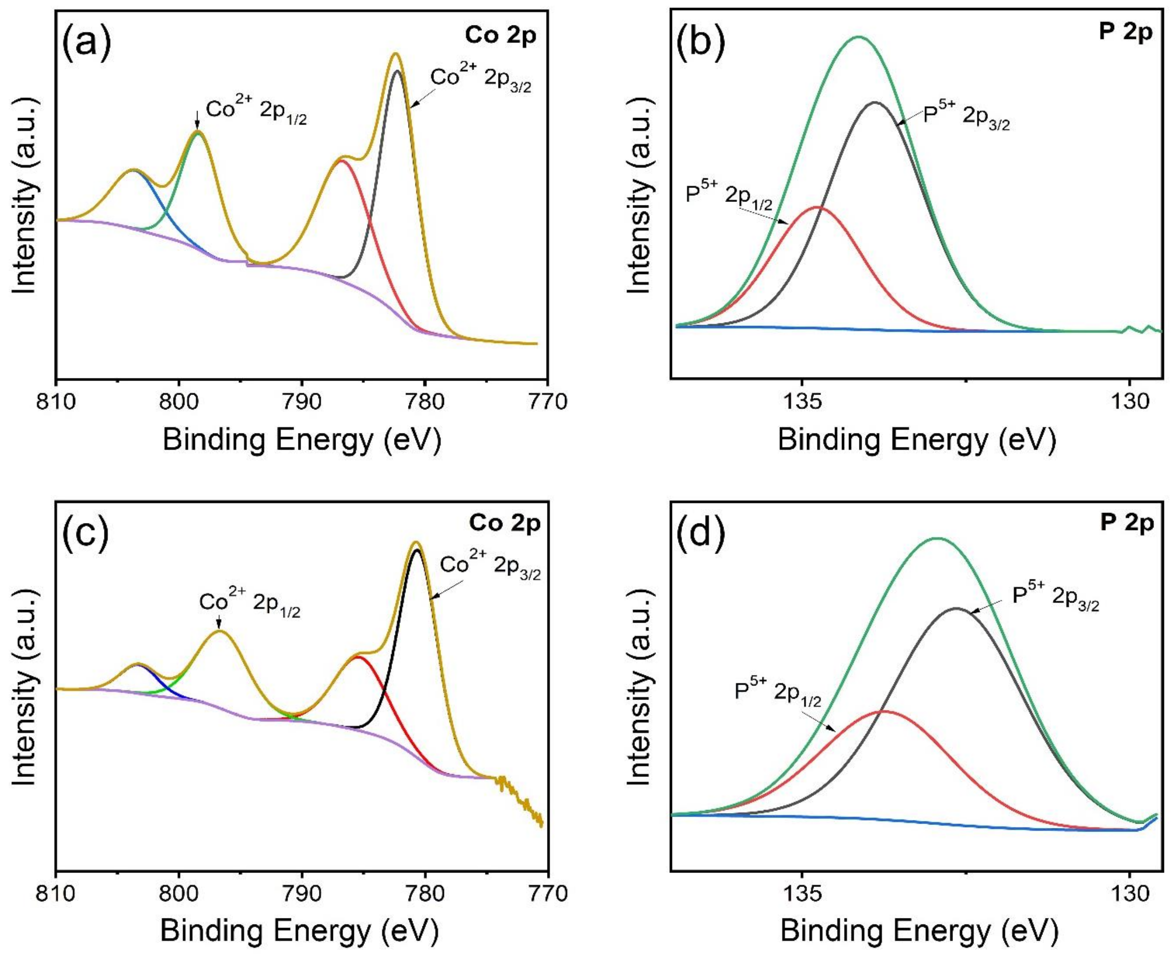

Figure S5. XPS spectra of Ni foam/CMP-350 before (a) Co 2p, (b) P 2p and after (c) Co 2p, (d) P 2p stability test (j = 10mA cm^{-2}, 48h).

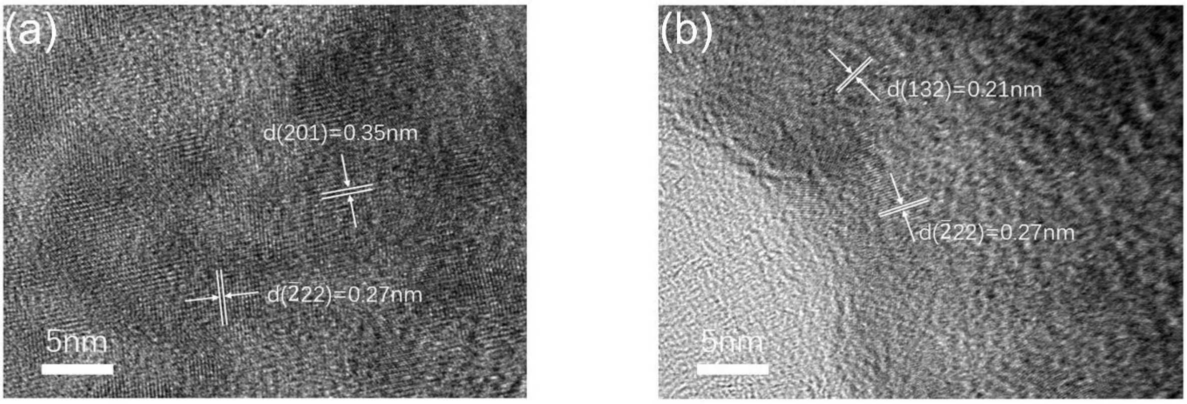

Figure S6. TEM images of Ni Foam/CMP-350 before (a) and after (b) stability test.

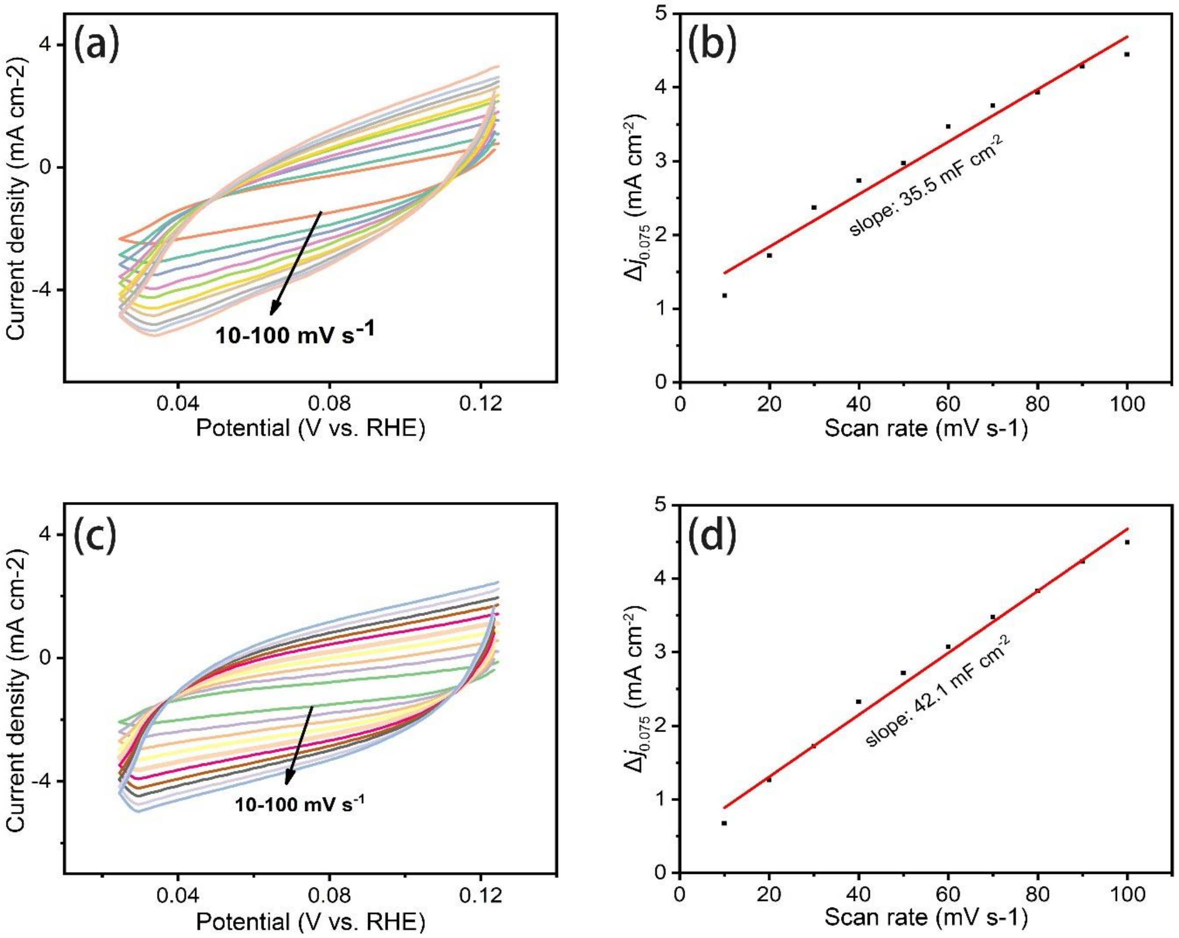

Figure S7. (a) CVs of Ni Foam/CMP-350 with different scan rates from 10 to 100 mV s-1. (b) The capacitive current at 0.075 V as a function of the scan rate for Ni Foam/CMP-350. (c) CVs of Ni Foam/CMP-500 with different scan rates from 10 to 100 mV s-1. (d) The capacitive current at 0.075 V as a function of the scan rate for Ni Foam/CMP-500.

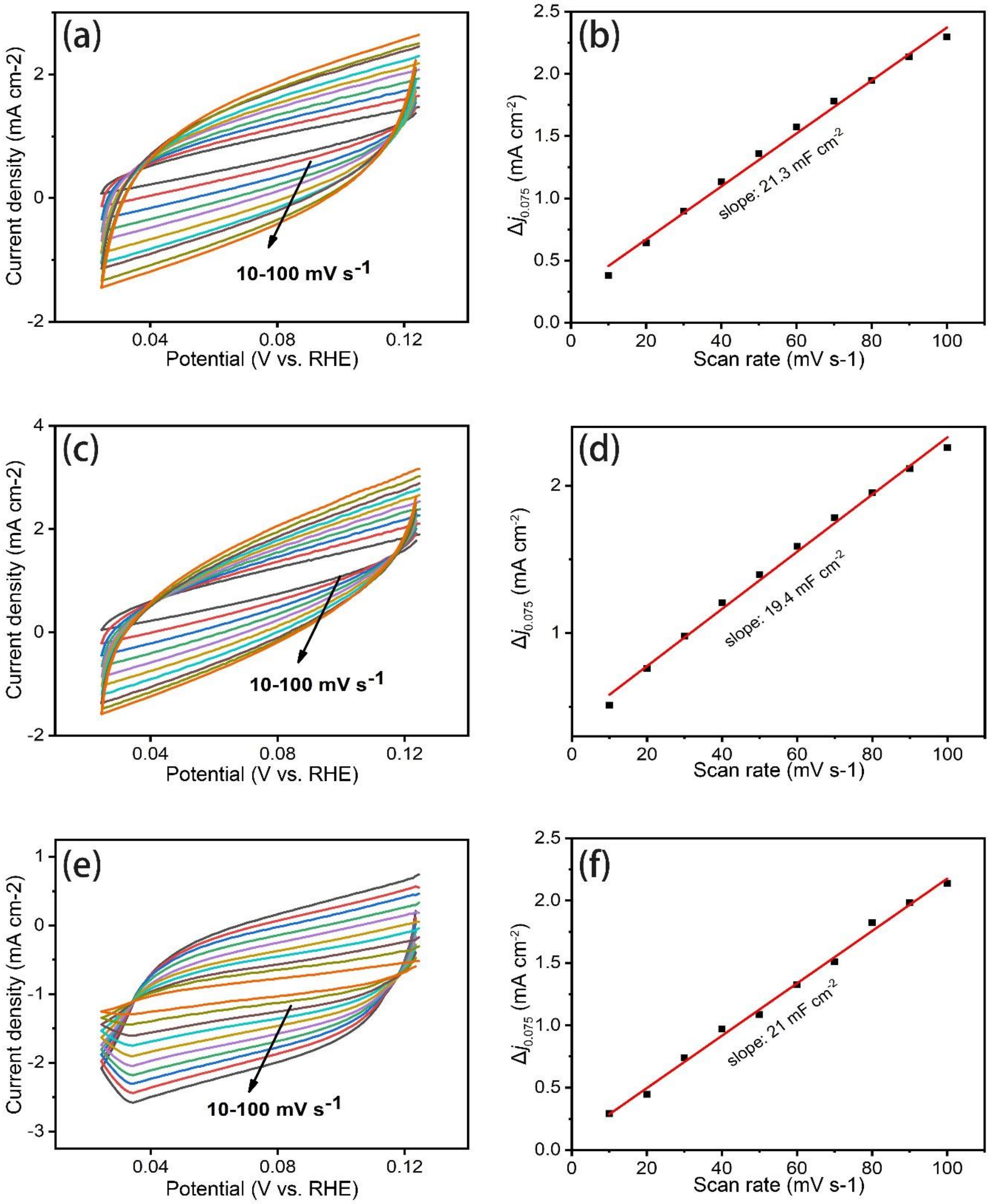

Figure S8. (a) CVs of Ni foam/CMP-300 with different scan rates from 10 to 100 mV s^{-1}. (b) The capacitive current at 0.075 V as a function of the scan rate for Ni foam/CMP-300. (c) CVs of Ni foam/CMP-400 with different scan rates from 10 to 100 mV s^{-1}. (d) The capacitive current at 0.075 V as a function of the scan rate for Ni foam/CMP-400. (e) CVs of Ni foam/CMP-450 with different scan rates from 10 to 100 mV s^{-1}. (f) The capacitive current at 0.075 V as a function of the scan rate for Ni foam/CMP-450.

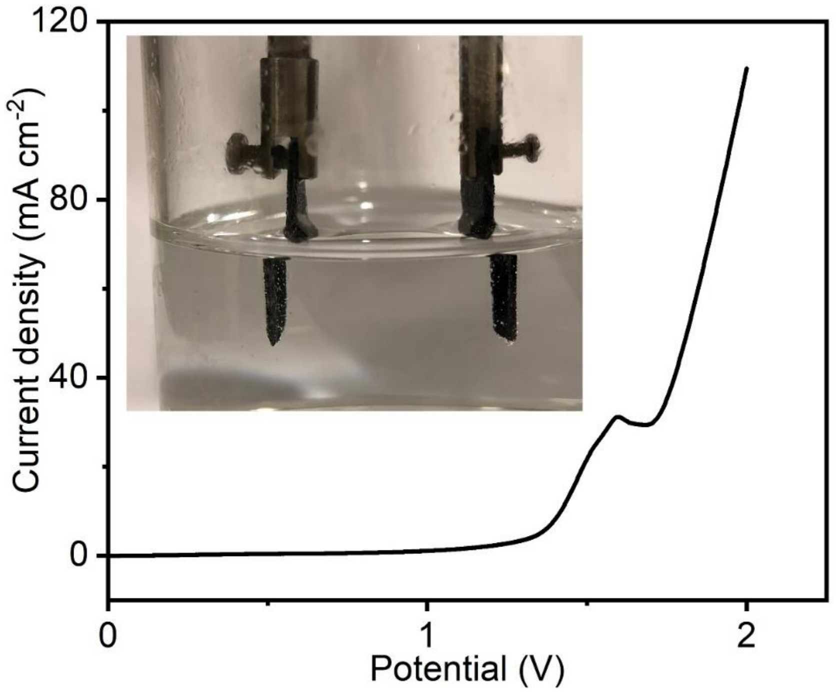

Figure S9. Polarization curve of Ni Foam/CMP-350 catalyst for overall water splitting in 1M KOH at a scan rate of 2mV/s. Ni Foam/CMP-350 electrodes were used as both working and counter electrodes. Inset: Graph of two-electrodes cell during reaction.

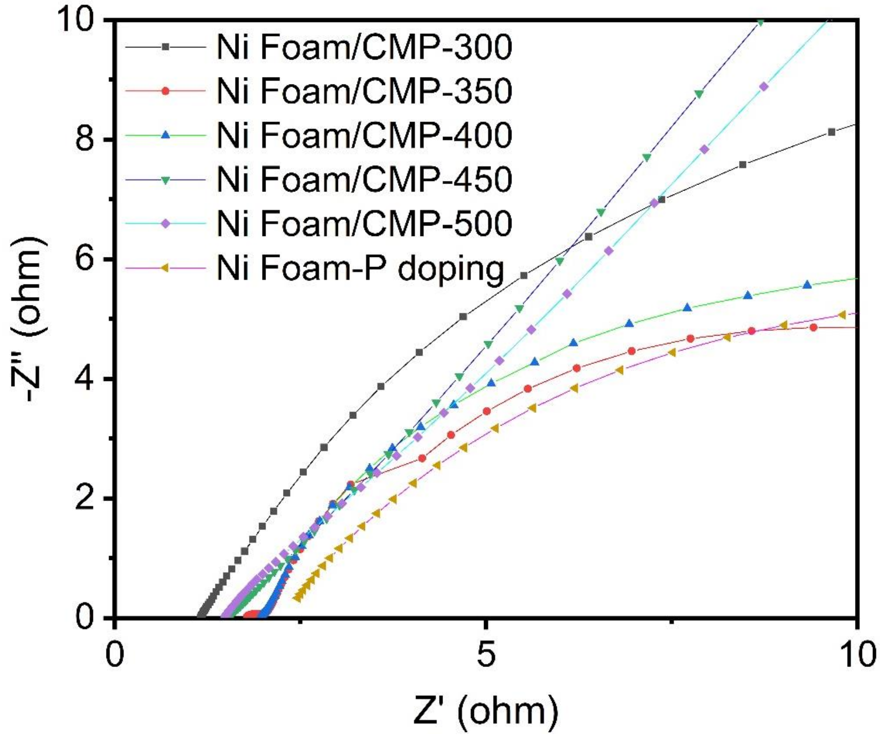

Figure S10. Nyquist plots of electrochemical impedance spectra (EIS) of sample electrodes recorded in 1M KOH solution.

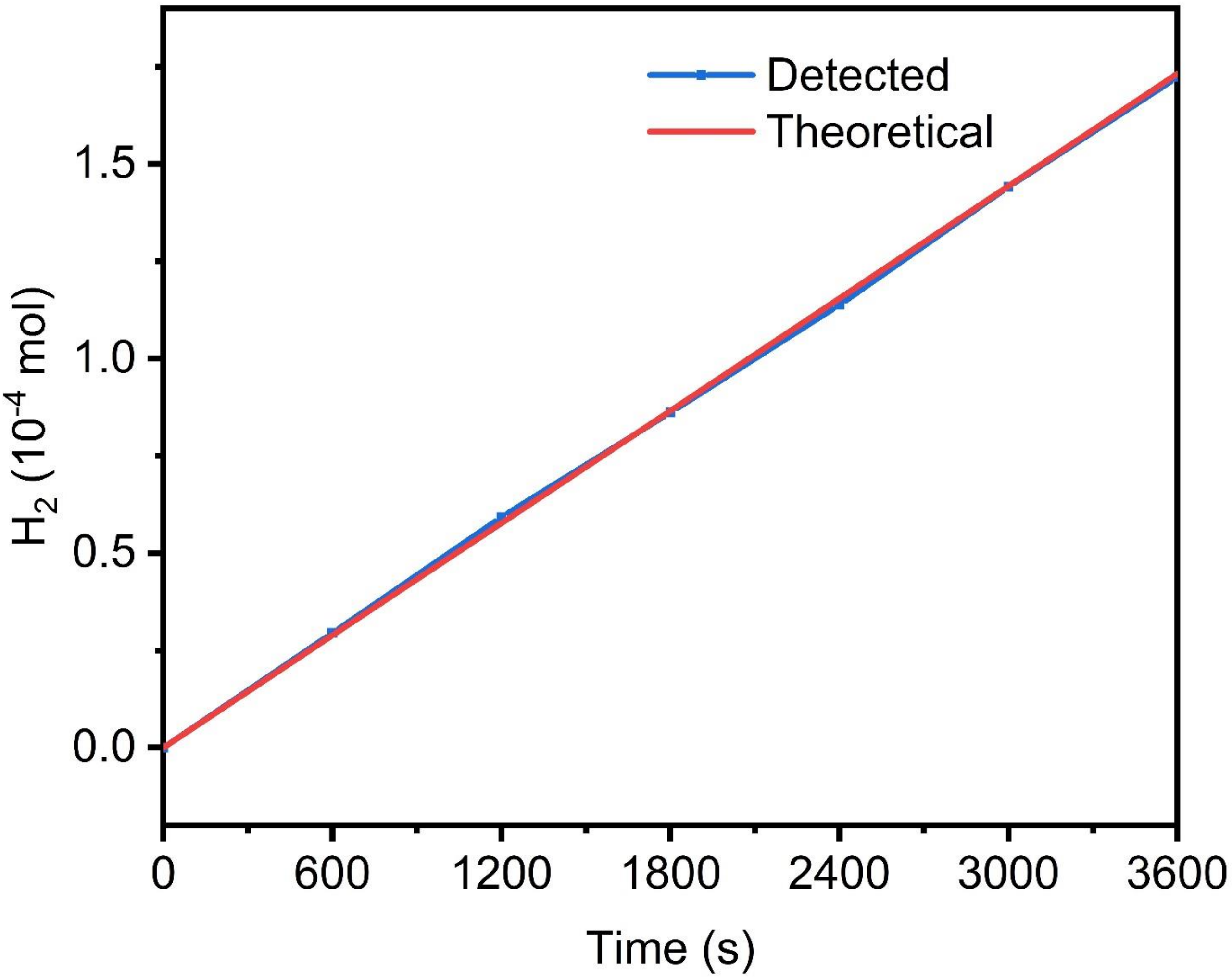

Figure S11. The theoretical (red) and detected (blue) values represent the expected and observed amount of H2 produced versus time for Ni foam/CMP-350.

Table S1. The XPS quantitative results of Ni foam/CMP electrodes prepared at different conditions.

Sample	Co:P
Ni foam/CMP-0.5h	1:3.63
Ni foam/CMP-2h	1:3.38
Ni foam/CMP-50mg	1:1.48
Ni foam/CMP-200mg	1:2.59
Ni foam/CMP-500mg	1:3.66
Ni foam/CMP-700mg	1:5.16

Table S2. Experimental and computational lattice parameters for β-$CoMoO_4$ and P-doped β-$CoMoO_4$. Computational and experimental values match well, and there is little overall change after P-doping.

	**a / Å**	**b / Å**	**c / Å**	**α / °**	**β / °**	**γ / °**
**$CoMoO_4$ Experimental**	10.21	9.27	7.022	90.0	106.9	90.0
**$CoMoO_4$ Computational**	10.26	9.31	7.07	90	107.4	90
**P-$CoMoO_4$ Computational**	10.14	9.29	6.97	90	107.0	90
